# Transcription factors, metabolic dysfunction-associated fatty liver disease, and therapeutic implications

**DOI:** 10.1016/j.gendis.2024.101372

**Published:** 2024-07-05

**Authors:** Shuwei Hu, Yingjie Ai, Chencheng Hu, Fathima N. Cassim Bawa, Yanyong Xu

**Affiliations:** a Department of Integrative Medical Sciences, Northeast Ohio Medical University, Rootstown, OH 44272, USA; b Department of Pathology of School of Basic Medical Sciences, Department of Gastroenterology and Hepatology of Zhongshan Hospital, Fudan University, Shanghai 200032, China; c Department of Pathology of School of Basic Medical Sciences, Fudan University, Shanghai 200032, China; d Institute of Diabetes, Obesity and Metabolism, Department of Medicine, University of Pennsylvania Perelman School of Medicine, Philadelphia, PA 19104, USA; e Key Laboratory of Metabolism and Molecular Medicine of the Ministry of Education, Frontier Innovation Center, Department of Pathology of School of Basic Medical Sciences, Fudan University, Shanghai 200032, China

**Keywords:** Apoptosis, Fibrosis, Inflammation, MAFLD, Steatosis, Transcription factors

## Abstract

Metabolic dysfunction-associated fatty liver disease (MAFLD) encompasses a spectrum of liver diseases ranging from metabolic dysfunction-associated fatty liver to metabolic dysfunction-associated steatohepatitis, which may progress to liver cirrhosis and hepatocellular carcinoma. Several mechanisms, including obesity, insulin resistance, dyslipidemia, inflammation, apoptosis, mitochondrial dysfunction, and reactive oxygen species, have been proposed to underlie the progression of MAFLD. Transcription factors are proteins that specifically bind to DNA sequences to regulate the transcription of target genes. Numerous transcription factors regulate MAFLD by modulating the transcription of genes involved in steatosis, inflammation, apoptosis, and fibrosis. Here, we review the pathological factors associated with MAFLD, with a particular emphasis on the transcription factors that contribute to the progression of MAFLD and their therapeutic implications.

## Introduction

Metabolic dysfunction-associated fatty liver disease (MAFLD) has emerged as a major global health issue in recent years, and its prevalence is increasing alongside the diabetes epidemic.^1^ Metabolic dysfunction-associated fatty liver (MAFL) and metabolic dysfunction-associated steatohepatitis (MASH) constitute the majority of MAFLD diseases.^2^ Currently, there are various intervention strategies for each stage of MAFLD; however, their reported efficacy is limited, and no effective strategy can simultaneously ameliorate hepatic steatosis and fibrosis progression. Although some reports appear to support the use of combination therapies for treating MASH,^2^, ^3^, ^4^, ^5^ their safety and side effects for combination trials must be carefully considered. Moreover, it is difficult to collect data on unforeseeable patient outcomes using combination therapies. Although numerous drugs and pharmacological therapeutic regimens have been evaluated in clinical trials, most have demonstrated inefficacy or are accompanied by intolerable adverse effects.

**Figure 1:**
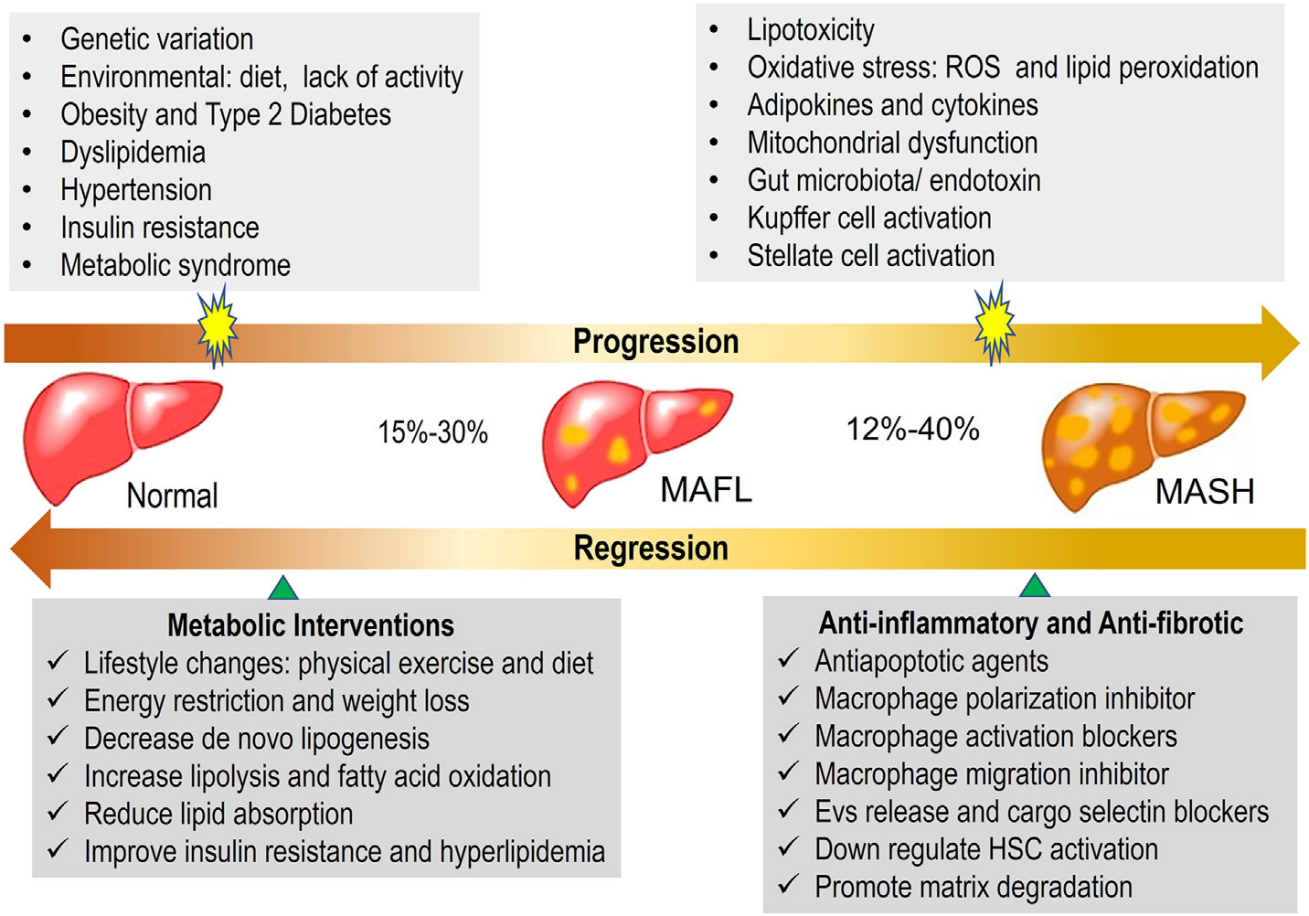
The MAFLD spectrum and treatment options for MAFL and MASH. There are some risk factors involved in the progression of MAFLD by mediating glucolipid metabolic disorders, Kupffer cell activation, stellate cell activation, *etc*. The currently effective treatment options for MAFL/MASH mainly include metabolic interventions and anti-inflammatory/anti-fibrotic treatment. MAFL, metabolic dysfunction-associated fatty liver; MAFLD, metabolic dysfunction-associated fatty liver disease; MASH, metabolic dysfunction-associated steatohepatitis.

To identify the ideal target and develop an effective treatment for MASH, it is necessary to understand its pathogenesis. As shown in Figure 1, MAFL and MASH are the two major types of MAFLD. In pathophysiological and etiological studies, a broad range of factors have been shown to be associated with MAFL progression, including genetic variation, environmental influences, such as diet or lack of physical activity, obesity, type 2 diabetes, dyslipidemia, hypertension, insulin resistance, and metabolic syndrome.^6^ In 1998, Day and James proposed a “two-hit hypothesis” to explain MAFLD pathogenesis.^7^ The first hit is mainly caused by insulin resistance, which results in the accumulation of triglycerides in the cytoplasm of hepatocytes, leading to MAFL formation. The worldwide prevalence of MAFL is approximately 15%–30%. On this basis, the second hit further causes hepatocellular injury, leading to MAFL progression to MASH in 12%–40% of cases.^8^ It is generally recognized that possible candidates for the second hit include lipo-toxicity, oxidative stress such as reactive oxygen species and lipid peroxidation, adipokines and cytokines, mitochondrial dysfunction, gut microbiota/endotoxin, Kupffer cell activation, and stellate cell activation. Specific intervention strategies are required for each stage to prevent the progression of MAFLD. For MAFL, metabolic interventions are directed toward lifestyle modifications, such as physical exercise and diet, energy restriction and weight loss, decreased *de novo* lipogenesis (DNL), increased lipolysis and fatty acid oxidation (FAO), reduced lipid absorption, and improved insulin resistance and hyperlipidemia.^9^ These strategies can stop or reverse the progression of MAFL; however, more effective approaches are needed to prevent or reverse the progression from MAFLD to MASH. Such approaches include anti-inflammatory and anti-fibrotic agents, anti-apoptotic agents, macrophage polarization inhibitors, macrophage activation blockers, macrophage migration inhibitors, extracellular vesicle release, and cargo selectin blockers, hepatic stellate cell (HSC) activation inhibitors, and matrix degradation promoters.^10^

Efforts to comprehend and elucidate the mechanisms controlling the complexity of MAFLD pathophysiology are now yielding positive results. Several studies have indicated that certain transcription factors play key roles in metabolic homeostasis, inflammatory and immune responses, apoptosis, and fibrogenesis during the pathogenesis and progression of MAFLD. Therefore, transcriptional factors appear to be appropriate therapeutic targets for hepatic steatosis and fibrosis in MAFLD. Indeed, drugs that modulate transcription factor function are currently undergoing MASH clinical trials.^11^ For example, the farnesoid X receptor (FXR) has emerged as one of the most promising drug targets for both hepatic steatosis and fibrosis.^12^ Obeticholic acid (OCA), as an FXR agonist, has demonstrated its potential to alleviate liver lipid accumulation, inflammation, and fibrosis in patients in a phase 3 clinical trial of anti-MASH.^13^ However, OCA has not been approved by the FDA due to an increased risk of cardiovascular disease.

Fortunately, resmetirom, as a nuclear resident transcription factor (THR-β) agonist, has recently received the FDA breakthrough therapy designation. THRs are transcription factors that bind to thyroid hormone and triiodothyronine, modulating the expression of several genes involved in DNL, FAO, gluconeogenesis, and cholesterol transport in the liver. Mice with a dominant negative mutation in THR-β (THR-βPV/PV) exhibit an MAFLD phenotype accompanied by hepatosteatosis.^14^ A similar THR-β mutation was found in humans, leading to a resistance to thyroid hormone phenotype, also resulting in an increase of hepatic fat content.^15^ Liver-specific THR-β agonists (resmetirom) as well as VK-2809/MB-07811 have shown significant efficacy in resolving MAFLD/MASH. More importantly, resmetirom significantly reduced hepatic steatosis and inflammation in patients with MASH in the pivotal phase 3 MAESTRO-NASH clinical trial with Madrigal Pharmaceuticals' oral MASH therapy.^16^^,^^17^

These studies indicate that transcription factors as drug targets can prevent MAFLD progression by modulating hepatic steatosis and fibrosis. Therefore, targeting transcription factors and developing specific drugs that can alleviate MAFLD symptoms without causing severe side effects are ideal strategies for treating MASH. In this review, we outlined the pathophysiology of MAFLD and highlighted the role of transcription factors in its management.

## Transcription factors in steatosis, inflammation, apoptosis, and fibrosis

### Steatosis

The development of MAFLD often initiates from steatosis. Hepatic steatosis is defined as more than 5% fat, with no evidence of hepatocellular injury in the form of ballooning of the hepatocyte cytoplasm. This is a key and essential histological feature of MAFL and is prevalent in human patients with insulin resistance, obesity, type 2 diabetes, and other factors that impair the balance of lipids metabolism.^18^^,^^19^ Multiple metabolic pathways lead to the formation of steatosis in hepatocytes, including increased DNL, decreased FAO, and enhanced fatty acid uptake (from adipose or intestine).^20^ Several studies have suggested that transcription factors are involved in the modulation of these metabolic pathways.

DNL is a process by which carbohydrates are converted into fatty acids, which are then used to synthesize triglycerides and other lipids.^21^ The transcriptional regulation of DNL is orchestrated by two key transcription factors, sterol regulatory element-binding protein 1c (SREBP1c) and carbohydrate response element-binding protein (ChREBP). SREBP-1c, a member of the transcription factor family (SREBP-1a, SREBP-1c, and SREBP-2), can be activated by insulin and liver X receptor α. Inactivation of SREBP-1c markedly improves hepatic steatosis by inhibiting DNL.^22^ FXR, a bile acid receptor, can lower hepatic triglyceride levels by increasing SHP (Src homology region 2 domain-containing phosphatase) expression, which can decrease the expression of SREBP-1c and DNL.^23^ Peroxisome proliferator-activated receptor α (PPARα) or Kruppel-like factor 15 (KLF15) can also ameliorate hepatic steatosis through inhibition of SREBP-1c activation to reduce the synthesis of fatty acids and triacylglycerols.^24^^,^^25^ In addition to SREBP-1c, ChREBP is another transcription factor that is highly expressed in organs that tend to accumulate fat, such as the liver, intestines, and adipose tissue, which is activated by carbohydrates and involved in DNL.^26^^,^^27^ Silencing ChREBP reduces triglyceride content in the liver, specifically through the inhibition of glucose-induced lipogenesis, whereas hepatic ChREBP overexpression leads to steatosis and increased DNL. Interestingly, zinc finger and BTB domain containing 20 (ZBTB20) can bind to and enhance the activity of the ChREBP-α promoter. ZBTB20 ablation protects against diet-induced liver steatosis and improves hepatic insulin resistance.^28^ X-box binding protein 1 (XBP1) is a transcription factor necessary for DNL in the liver. Inhibition of XBP1 in hepatocytes alleviates liver steatosis and MASH.^29^

Hepatic steatosis is controlled by FAO and fatty acid uptake. Activation of PPARα up-regulates the transcription of a range of genes related to FAO in hepatocytes, thereby reducing hepatic steatosis. In contrast, knockout of PPARα in mice results in hepatic steatosis. Some transcription factors can modulate FAO by coordinating with PPARα; for example, FXR can interact with PPARα in hepatocytes and enhance PPARα-mediated FAO.^30^ In addition to PPARα, hepatocyte nuclear factor 4α (HNF4α) is also an essential regulator of hepatic FAO. Overexpression of hepatocyte HNF4α promotes lipolysis and FAO by up-regulating the expression of triglyceride hydrolysis-related enzymes (CES1 and CES2) or p53, whereas loss of hepatocyte HNF4α has the opposite effect.^31^ Interestingly, our previous studies illustrated that hepatocyte activating transcription factor (ATF3) plays a preventative role in the progression of MAFL to MASH by modulating FAO through HNF4α.^32^ Moreover, uptake of fatty acids beyond the metabolic requirements in hepatocytes results in the accumulation of triglycerides and hepatic steatosis; for example, activation of pregnane X receptor (PXR) increases hepatocyte lipid uptake and leads to steatosis by up-regulating the fatty acid transporter CD36.^1^, ^2^, ^3^, ^4^

In addition to regulating lipid metabolism, transcription factors that regulate glucose metabolism or endoplasmic reticulum stress can also modulate hepatic steatosis. For example, the constitutive androstane receptor (CAR), a member of the nuclear receptor superfamily involved in glucose metabolism, plays a crucial role in diabetes and fatty liver disease.^5^ CAR alleviates high-fat diet (HFD)/phytic acid-induced steatosis and liver injury. The treatment of genetically obese mice with a CAR agonist improved glucose tolerance and insulin resistance, resulting in attenuated hepatic steatosis.^6^ In contrast, inhibition of CCAAT enhancer binding protein alpha (C/EBPα) or Yin Yang 1 (YY1) activation can improve glucose metabolism and hepatic steatosis.^7^ Moreover, certain transcription factors regulated by endoplasmic reticulum stress play critical roles in hepatic steatosis. In response to endoplasmic reticulum stress, activating transcription factor 6α (ATF6α) can attenuate liver steatosis by stimulating PPARα-mediated FAO. Hypoxia-inducible factor-1α (HIF-1α) can also attenuate oxidative stress-induced lipid accumulation in the development of MAFLD.^8^ Conversely, XBP1 increases the incidence of steatosis and MASH in response to endoplasmic reticulum stress.

**Figure 2:**
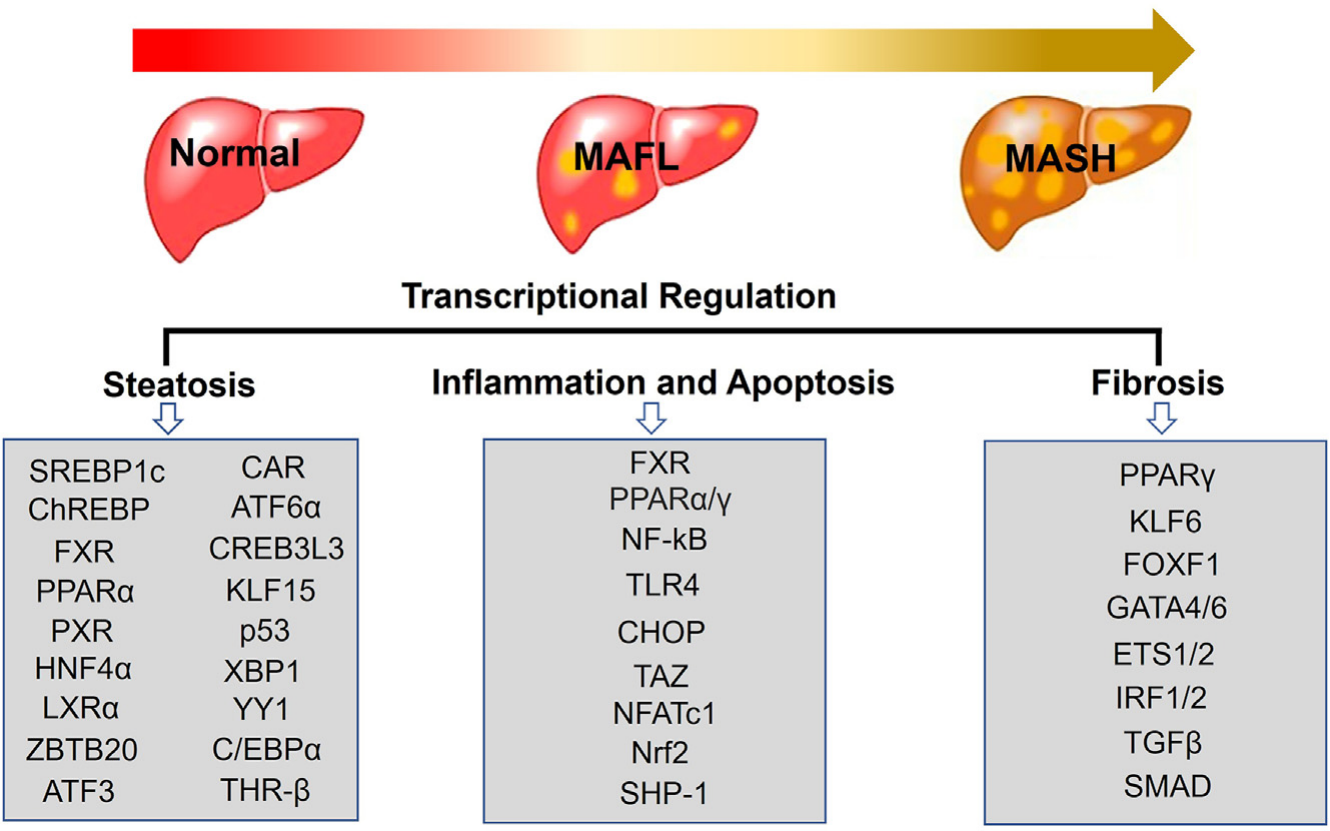
Transcriptional regulation of steatosis, inflammation, apoptosis, and fibrosis in the progression of MAFLD. ATF3, activating transcription factor 3; ATF6α, activating transcription factor 6 alpha; CAR, constitutive androstane receptor; C/EBPα, CCAAT enhancer binding protein alpha; CHOP, C/EBP-homologous protein; ChREBP, carbohydrate response element binding protein; CREB3L3, CAMP responsive element binding protein 3 like 3; FOXF1, forkhead box F1; GATA4/6, FXR, farnesoid X receptor; GATA binding protein 4; HNFα, hepatocyte nuclear factor 4 alpha; IRF1/2, interferon regulatory factor 1/2; KLF15, Kruppel like factor 15; KLF6, Kruppel like factor 6; LXRα, liver X-activated receptor alpha; MAFLD, metabolic dysfunction-associated fatty liver disease; NFATc1, nuclear factor of activated T cells 1; NF-kB, nuclear factor kappa-B; Nrf2, nuclear factor erythroid derived 2-like 2; p53, cellular tumor antigen p53; PPARα/γ, peroxisome proliferator activated receptor alpha/gamma; PXR, pregnane X receptor; SHP-1, Src homology region 2 domain-containing phosphatase 1; SMAD, drosophila mothers against decapentaplegic protein; SREBP1C, sterol regulatory element binding protein-1c; TAZ, transcriptional coactivator with PDZ-binding motif; TGFβ, transforming growth factor beta; THR-β, thyroid hormone receptor beta; TLR4, Toll-like receptor 4; XBP1, X-box binding protein 1; YY1, Yin and Yang 1 protein; ZBTB20, zinc finger and BTB domain containing 20.

In summary, transcription factors modulate hepatic steatosis through multiple mechanisms. A significant number of transcription factors are involved in the modulation of the progression of hepatic steatosis and MAFL, such as SREBP1c, ChREBP, FXR, PPARα, PXR, HNF4α, liver X-activated receptor alpha (LXRα), ZBTB20, ATF3, CAR, ATF6α, CAMP responsive element binding protein 3 like 3 (CREB3L3), KLF15, p53, XBP1, YY1, C/EBPα, and THR-β (Fig. 2).

### Inflammation and apoptosis

MASH is an advanced stage of MAFLD characterized by steatosis, liver cell injury, and inflammation, which may further progress to fibrosis and cirrhosis.^9^ Hepatic inflammation and apoptosis are induced by various factors that cause a disproportionate loss of hepatocytes, leading to irreversible liver damage, fibrosis, and carcinogenesis. Kupffer cells, HSCs, sinusoidal endothelial cells, and cells recruited in response to injury, such as monocytes, macrophages, dendritic cells, and natural killer cells, emit pro-inflammatory signals that result in hepatocyte apoptosis or necrotizing.^10^ In this section, we discuss transcription factors associated with liver inflammation and apoptosis.

In addition to regulating hepatic lipid/lipoprotein metabolism, gluconeogenesis, glycogen synthesis, and insulin sensitivity, activated FXR pathway improves liver inflammation and fibrosis.^11^ Similarly, activating PPARα/γ in MASH has also shown protective roles in anti-steatosis, anti-inflammatory, and anti-fibrotic effects.^12^ For example, PPARα can reduce liver inflammation by increasing the expression of leukotriene B4 (LTB4), a catabolic enzyme that inhibits extracellular LTB4-mediated inflammation and prevents nuclear factor kappa B (NF-κB)-mediated up-regulation of interleukin (IL)-6 and IL-12 ^13^. Interestingly, the regulation of inflammation by transcription factors can affect lipid metabolism during MASH progression. For example, Toll-like receptor 4 (TLR4) can exacerbate lipid metabolism disorders in MASH by interacting with fibrinogen-like protein 2 (FGL2)^14^ and activating XBP1,^15^ which leads to the generation of reactive oxygen species.^16^ Activation of NF-κB induces the expression of pro-inflammatory cytokines in macrophages, resulting in the dysfunction of lipid metabolism and aggravating the progression of MASH.^17^ Nuclear factor of activated T cells 1 (NFATc1) promotes MAFLD progression by increasing hepatic inflammation and fibrosis.^18^ Silencing the expression of TAZ (transcriptional coactivator with PDZ-binding motif) in hepatocytes could reverse inflammation, cell death, and fibrosis in NASH.^19^^,^^20^ Other than TLR4, NF-κB, NFATc1, and TAZ, SHP-1, nuclear factor erythroid-derived 2-like 2 (Nrf2), and LXRα are negative regulators of inflammation. SHP-1 improves steatohepatitis by inhibiting the production of pro-inflammatory cytokines, transforming growth factor-β (TGF-β), IL-6, and tumor necrosis factor alpha (TNFα).^21^

Inhibition of the phosphorylation of LXRα attenuates inflammation and fibrosis during MAFLD progression.^22^ Nrf2 regulates gene expression involved in anti-oxidation,^23^ and stimulation of Nrf2 signaling pathway can protect the liver from oxidative injury.^24^ Apoptosis has evolved as a critical mechanism contributing to the progression of MAFLD from benign simple steatosis to the more aggressive form of MASH. CCAAT/enhancer-binding protein homologous protein (CHOP) is a marker of endoplasmic reticulum stress and plays a key role in endoplasmic reticulum stress-induced apoptosis.^25^ Under fat-loading conditions, the absence of CHOP reduces fat-induced apoptosis, inflammation, and fibrosis.^26^ Loss of CHOP in mice reduces hepatocyte apoptosis by down-regulating the expression of alpha-smooth muscle actin (α-SMA) and TGF-β1 ^27^.

Collectively, inflammation and apoptosis are pathological hallmarks of MASH, positively correlated with disease severity, and significantly induced in patients and animals with MASH. Transcription factors involved in the regulation of inflammation and apoptosis during MASH progression include FXR, PPARα/γ, NF-kB, TLR4, CHOP, TAZ, NFATc1, Nrf2, and SHP-1 (Fig. 2).

### Fibrosis

The severity of hepatic fibrosis is the main histological predictor of liver-related morbidity and mortality in patients with MASH.^28^ HSCs represent the major fibrogenic cell population responsible for most architectural changes characterizing fibrotic or cirrhotic MASH livers. HSC activation, proliferation, and fibrogenic myofibroblasts are established as central drivers of hepatic fibrosis in experimental and human MASH.^29^ Phenotypes of HSCs from humans and mice are regulated by transcription factors, including PPARγ, Kruppel-like factor 6 (KLF6), forkhead box f1 (FOXF1), GATA binding protein 4/6 (GATA4/6), E-twenty-six 1/2 (ETS1/2), interferon regulatory factor 1/2 (IRF1/2), TGF-β, and SMAD (drosophila mothers against decapentaplegic protein). PPARγ and KLF6 are required for the inactivation of human HSCs and regression of liver fibrosis in mice.^30^^,^^31^ Activation of PPARγ attenuates the development of MASH by reducing HSC proliferation, down-regulating extracellular matrix gene expression, and restraining TGF-β1 signaling. Early induction of KLF6 during HSC activation can decrease fibrogenic activity by transcriptionally repressing target genes and increasing apoptosis of activated HSCs. In contrast, FOXF1 is strongly associated with HSC activation.^32^ FOXF1 knockdown blocks HSC activation, reducing liver injury and fibrosis in mice.^33^ Moreover, GATA4/6, IRF1/2, and ETS1/2, which are HSC lineage-specific transcription factors in mouse and human HSCs, are associated with liver fibrosis. Inactivation of these transcriptional factors in HSCs induces the expression of fibrogenic and inflammatory genes, resulting in the loss of their lineage-specific phenotype. Transcription factors-controlled cellular signals play a critical role in HSC activation. TGF-β triggers liver fibrosis by inducing hepatocyte epithelial–mesenchymal transition. Various studies have demonstrated that suppressing TGF-β signaling could ameliorate liver fibrosis.^34^^,^^35^ Transcriptional modulators SMADs play crucial roles in the transmission of TGF-β signals from cell surface receptors to the nucleus, and different SMADs mediate signal transduction of various TGF-β family members. SMAD2 and SMAD7 protect against hepatic fibrosis, whereas SMAD3 and SMAD4 are pro-fibrotic. Targeting the SMAD signaling pathway can regulate HSC activation, thus attenuating liver fibrosis.^36^

Targeting the development of hepatic fibrosis is a key strategy for preventing the progression of MAFL to MASH or liver cirrhosis. Identification of transcription factors that play an important role in hepatic fibrosis is essential for transcription factor-based drug development for MASH.

## Related drug studies in modulation of transcription factors in MAFLD

### THR

THRs include two different isoforms, namely THR-α and THR-β, primarily involved in the metabolism of cholesterol and lipoproteins, and may serve as possible targets for antifibrotic therapy.^37^ It has been reported that the expression of THR-α and THR-β was decreased in liver tissue during liver disease. Current research focuses mostly on liver tissue-specific THR-β activators due to the increase in cholesterol absorption and synthesis in hepatocytes, which offers an ideal therapy option for MAFLD and MASH. The selective THR-β agonist Resmetirom (MGL-3196) mediates the increase of sterol efflux from liver and bile.^38^ VK2809 is a novel liver directed THR agonist that provides a durable reduction in liver fat in patients with NASH.^39^ THR-α dominates HSCs and may harbor a key role in HSC cell differentiation, while nonspecific THR activation has been shown to be deleterious, as activation of the THR-α receptor may lead to cardiotoxicity.^40^

### FXR

FXR, a bile acid-activated nuclear receptor, is mainly expressed in the liver and intestine. However, the metabolic roles of FXR in the intestine and liver differ. Intestinal FXR antagonism enhances metabolic regulation, whereas liver FXR activation improves hepatic fibrosis.^41^ Hepatic FXR not only prevents steatosis but also ameliorates inflammation and fibrosis. FXR agonists are developed for the treatment of MASH because of their inhibitory effects on lipogenesis and hepatic fibrosis.^42^ Mechanistically, FXR inhibits hepatic fatty acid and triglyceride synthesis by down-regulating the expression of SREBP1c and increasing hepatic FAO by up-regulating pyruvate dehydrogenase kinase. As a potential therapeutic target, FXR has been used extensively in clinical trials. In phase III clinical trials, OCA was a strong FXR agonist. Although FXR exerts hepatoprotective effects, it ameliorates liver steatosis, inflammation, and fibrosis. However, OCA treatment of MASH-induced liver fibrosis has an extremely low response rate and causes adverse symptoms such as itching.

Based on known preclinical findings and clinical applications, oral intestinal FXR inhibitors prioritize improving glucose and cholesterol metabolism, whereas liver targeted FXR agonists will improve liver function and fibrosis.^43^^,^^44^ In the future, it will be important to highlight whether the existing unknown interferes with the FXR signaling pathway between the liver and intestine, which may generate harmful consequences eventually.

### PPARα/γ

PPARα and PPARγ belong to non-steroid hormone receptors. Systemic or liver-specific knockout of PPARα aggravates hepatic steatosis and obesity induced by HFD or MASH diet in mice.^45^^,^^46^ Activation of PPARα using fibrate medications (clofibrate and fenofibrate) to treat fatty liver is performed in clinical intervention trials.^47^^,^^48^ One study reported considerable improvement in the distribution of lipids in individuals with MAFLD after treatment with fenofibrate, including reduced serum levels of alanine aminotransferase (ALT) and aspartate transferase (AST); however, interestingly, the liver histology remained unchanged.^48^ In a separate trial, ALT, AST, glycoprotein gamma-glutamyltransferase, bilirubin, triglycerides, cholesterol, and histology-graded steatosis, inflammation, or fibrosis showed improvement in fenofibrate-treated individuals; nevertheless, some patients experienced adverse effects and discontinued treatment.^47^

In HFD-induced or obese mouse models, liver-specific knockout of PPARγ was able to protect mice from fatty liver.^49^^,^^50^ PPARγ agonists (rosiglitazone and pioglitazone) also improve fatty liver disease in patients with MASH. Rosiglitazone decreases steatosis and AST levels but causes weight gain in patients.^51^ Despite no reduction in liver fibrosis or weight gain, treatment with pioglitazone resulted in metabolic and histological benefits in patients with MASH.^52^^,^^53^

In addition to agonists for specific subtypes, pharmacological double PPARα/γ agonists that activate more than one PPAR, called glitazars, have been developed to improve insulin resistance, dyslipidemia,^54^ and fatty liver^55^ in rodents. Saroglitazar has been demonstrated to dramatically improve liver fat content, ALT, insulin resistance, and atherosclerotic dyslipidemia in patients with MAFLD/MASH, as well as increase lipoprotein particle structure and size, thereby reducing the number of lipotoxic lipids.^56^ However, it has been proven that several chemicals in this class of medications can cause cardiovascular and kidney diseases.^57^

### LXR

LXRs include two different isoforms, LXR-α and LXR-β. They are also involved in the regulation of liver inflammation, fibrosis, cholesterol metabolism, and hepatic steatosis. LXR expression was closely associated with the degree of hepatic fat deposition, liver inflammation, and fibrosis in MAFLD.^58^ LXRs exert an anti-inflammatory effect,^59^ increase cholesterol efflux,^60^ and promote lipid production^61^ and gluconeogenesis.^62^ In hepatocytes, LXR-α activation enhances lipogenesis and results in the development of fatty liver disease. In the non-parenchymal cells of the liver, LXR-α agonism promotes the re-differentiation of primary liver sinusoidal endothelial cells, which alleviates liver inflammation and promotes the regression of liver fibrosis.^63^

Huang et al reported that the LXR inverse agonist SR9243 effectively inhibited liver fibrosis in a NASH mouse model.^64^ The LXR synthetic inhibitor SR9238 is also effective in reducing hepatic steatosis, inflammation, and fibrosis in animal models of MASH.^65^ Because LXR agonists, inverse agonists, and LXR synthetic inhibitors are all favorable for liver fibrosis, it is unclear whether LXR modulates direct or indirect antifibrotic actions. It is important to note that LXR-α activation produces severe adverse effects, such as hyperlipidemia and hepatic steatosis. Given that activation of LXR-α induced hepatic steatosis, studies targeting LXR regulation should focus on the modulation of LXR-β in the future.

### ATF3

ATF3 is a member of the ATF/cAMP response element-binding family that regulates gene transcription by binding to target gene promoters.^66^ ATF3 is associated with lipid and bile acid metabolism and inflammation. ATF3 is a potential yet debatable therapeutic target for MAFLD.

The function of ATF3 in lipid metabolism is still a matter of debate. ATF3 is a critical regulator of high-density lipoprotein cholesterol and bile acid metabolism in hepatocytes. Hepatocyte ATF3 improves high-density lipoprotein uptake, decreases intestinal lipid absorption, and stimulates macrophage reverse cholesterol transport.^67^^,^^68^ ATF3 knockout mice fed a high fat or chow diet gained weight more rapidly. Sulfuretin, a phytochemical ATF3 inducer, reduced adiposity in HFD-fed obese mice.^69^ Another ATF3 inducer isolated from *Salvia miltiorrhiza*, ST32da, also ameliorates obesity and suppresses adipogenesis/lipogenesis-related genes by multiple pathways, such as the carbohydrate-responsive element-binding protein–stearoyl-CoA desaturase-1 axis, C/EBPα, and the CHOP pathway.^70^^,^^71^ However, other studies have suggested that ATF3 promotes hepatic steatosis. By decreasing ATF3, cardiolipin synthase 1 (CRLS1), a member of the cytidine diphosphate-alcohol phosphatidyl transferase class-I family, decreases insulin resistance, hepatic steatosis, inflammation, and fibrosis.^72^ ATF3 knockdown in Zucker diabetic fatty rats also ameliorates hepatic steatosis by restoring FAO.^73^

In addition to its role in lipid metabolism, ATF3 also inhibits inflammatory reactions. ATF3 interacts with and inhibits p65, a component of the NF-kB dimer. After HDAC1 is recruited to the ATF3/p65 complex, p65 is deacetylated and its translocation is reduced, thereby reducing the activation of inflammatory response genes.^74^ To date, only a few drugs targeting ATF3 have been developed.

## Conclusions and perspectives

Gene amplification, mutations, genetic instability, epigenetics, and post-transcriptional alterations can lead to aberrant gene expression or function, which are the primary causes of MAFLD. Although some of these approaches have provided promising results and have been provisionally licensed as innovative medicines, numerous studies on transcription factors in liver disease have been conducted, but their long-term efficacy, tolerance, and safety must be investigated further. To further investigate and develop effective targeted therapies for liver-related diseases, it is necessary to pay more attention to and target transcription factors that improve lipid accumulation, fibrosis, and circulatory system homeostasis without significantly disrupting the circulatory system.

Considering the numerous transcription factors related to MAFLD, it is essential to identify the transcription factors that play a greater role in the progression of MAFLD. THR, FXR, PPARα/γ, and LXR regulate the development of MAFLD, and corresponding drugs are also undergoing clinical trials and have partly achieved positive results. For example, THR has a promising future in the treatment of MASH by modulating energy metabolism, liver inflammation, and fibrosis. THR-β is the main thyroxine receptor of the liver, which can mediate cholesterol metabolism and excretion through bile acid. High selectivity of the THR-β agonist MGL-3196 has been developed to treat dyslipidemia. PPARα/γ decreases fatty acid synthesis, regulates inflammation, and prevents the activation of HSCs. Therefore, it would be wise to target these two transcription factors simultaneously, as in the case of Saroglitazar. LXRs exert anti-inflammatory effects, boost cholesterol efflux, and stimulate lipid synthesis and gluconeogenesis. Consequently, the therapeutic efficacy of MAFLD is debatable due to its metabolic effects. ATF3 is a transcription factor with several functions, and accumulating evidence has linked it to glucolipid metabolism. Despite the disagreement regarding its effect, it is plausible to anticipate the development of comparable medications.

Patients, regulatory authorities, biotechnology and pharmaceutical businesses have shown a great deal of interest in the treatment of MAFLD and MASH owing to their increasing influence on global health. Despite the small number of clinical trials conducted to date, it is possible to improve MASH-related histology with medicines. However, more powerful and long-lasting effects of pharmacological therapy and reduced side effects must be established and connected to superior clinical outcomes, such as decreased liver inflammation and advanced liver fibrosis. Ideally, these medicines could ameliorate the characteristics of metabolism-related disorders, such as cardiovascular disease, which remains the leading cause of death in individuals with MAFLD, including those with MASH.

Identifying highly specific medications for the treatment of MAFLD is important for future therapeutic applications. Therefore, further research and development is required before transcription factor medications can be used. Targeting the activity of transcription factors is an emerging field with promising applications. Further research on the molecular underpinnings of transcription factor function could lead to the development of medications that fine-tune specific transcription factor functions for therapeutic purposes.

## Author contributions

S.H. drafted the first manuscript. S.H. and Y.X. checked and revised the manuscript. Y.A., C.H., and F.N.C.B. participated in the review design and helped modify the manuscript. All authors read and approved the final version of the manuscript.

## Conflict of interests

The authors declared no competing interests.

## Funding

This work was supported by the 10.13039/501100001809National Natural Science Foundation of China (No. 32271218 to Y.X.).
